# Exploring Exercise Addiction, Self-Esteem, and Early Maladaptive Schemas: A Cross-Sectional Study Among Female University Students

**DOI:** 10.3390/healthcare13040422

**Published:** 2025-02-15

**Authors:** Leticia Olave, Itziar Iruarrizaga, Patricia Macía, Janire Momeñe, Ana Estévez, José Antonio Muñiz, Cecilia Peñacoba

**Affiliations:** 1 Department of Experimental Psychology, Cognitive Processes and Speech Therapy, Faculty of Social Work, Complutense University of Madrid, 28223 Madrid, Spain; leticiaolave@ucm.es (L.O.); iciariru@psi.ucm.es (I.I.); jomuniz@ucm.es (J.A.M.); 2 Department of Basic Psychological Processes and Their Development, University of the Basque Country, 20018 Donostia-San Sebastián, Spain; patricia.macia@ehu.eus; 3 Department of Psychology, Faculty of Health Science, University of Deusto, 48007 Bilbao, Spain; janiremomene@deusto.es (J.M.); aestevez@deusto.es (A.E.); 4 Department of Psychology, Faculty of Health Sciences, Universidad Rey Juan Carlos, 28933 Madrid, Spain

**Keywords:** exercise addiction, self-esteem, early maladaptive schemas, women

## Abstract

Background/Objectives: Although physical exercise provides numerous health benefits, it can occasionally become addictive, leading to negative consequences for physical and mental health. Specifically, the role of maladaptive schemas in the relationship between exercise addiction and self-esteem underscores the importance of addressing these cognitive patterns in therapeutic settings to develop practical interventions that enhance exercise with healthier self-perceptions. This study aims to analyze the role of early maladaptive schemas in the relationship between exercise addiction and self-esteem. Methods: The design of this study is non-experimental, correlational, and cross-sectional. The sample comprised 788 university women students (mean age 20.39 years, SD = 2.28). Results: Exercise addiction is negatively associated with self-esteem and shows positive but weak correlations with most early maladaptive schemas, except for Impaired Autonomy. A mediating effect was identified for Disconnection and Rejection (β = −0.08, *p* = 0.008), Impaired Limits (β = −0.03, *p* = 0.019), Other Directedness (β = −0.04, *p* = 0.032), and Over-Vigilance and Inhibition (β = −0.05, *p* < 0.001). Full mediation was observed for Disconnection and Rejection and Over-Vigilance and Inhibition, while Impaired Limits and Other Directedness showed partial mediation. Conclusions: These findings suggest that the decrease in self-esteem among individuals with exercise addiction could be explained by the activation of maladaptive schemas that influence exercise motivation, with Over-Vigilance and Inhibition standing out in particular. Furthermore, it is necessary to develop cognitive behavioral therapy (CBT)-based interventions focused on modifying early maladaptive schemas and strengthening self-esteem. Additionally, it would be advisable to implement educational programs in university and sports settings that promote well-being and enjoyment over the pursuit of external validation or obsession with body image. These strategies could help prevent exercise addiction and mitigate its negative effects on self-esteem.

## 1. Introduction

Physical exercise offers innumerable benefits for the biopsychosocial adjustment of humans, contributing to improved physical and mental health [1]. Physical activity has been associated with reduced depressive and anxious symptoms, better sleep quality, and improved quality of life [2]. Additionally, exercise enhances cognitive function both in the general population and in individuals with symptoms of psychotic spectrum disorders and bipolar disorder [3]. It also has a positive impact on executive functions in children, reducing symptoms associated with attention-deficit disorder [4].

Conversely, individuals who do not engage in sufficient physical activity have a 20% to 30% higher risk of mortality compared to those who maintain adequate levels of activity [5], with physical inactivity being the fourth leading risk factor for global mortality [6].

Notably, one of the well-documented benefits of physical activity on emotional well-being is its positive impact on self-esteem [3,7,8,9,10]. Positive effects of physical exercise on self-esteem have been observed in adolescent populations, enhancing self-concept [11,12] and body image perception [10]. These effects have also been documented in adult and older adult populations [13] across both men and women [14,15,16].

Despite the numerous health benefits of physical activity, when exercise becomes compulsive and individuals lose control over their behavior, it can develop into an addictive pattern [17,18]. Behavioral addiction to physical exercise develops through positive reinforcement and it is maintained via negative reinforcement [17]. It can be assessed by identifying symptoms commonly associated with substance addictions, such as tolerance, withdrawal, desired effects, lack of control, time investment, reduction in other activities, and persistence of behavior [19,20,21].

The relationship between exercise addiction and self-esteem is underexplored, but some studies suggest that exercise addiction is associated with lower self-esteem [22,23,24,25,26]. Low self-esteem may act as a predictor of exercise addiction, sometimes alongside other variables, such as narcissistic traits [27], emotional dependence, and impulsivity [28]. This aligns with evidence indicating that deficient self-esteem underpins addictive disorders, whether related to substances or behaviors, serving as a risk factor for the initiation and maintenance of addiction [29,30,31,32]. Conversely, high self-esteem could be proposed as a protective factor against the development of exercise addiction symptoms [25], similar to its protective role in other behavioral addictions, such as internet gaming addiction [33].

Another fundamental psychological variable related to mental health is the concept of early schemas. These are patterns or internalized cognitive schemas that are stable and acquired through early affective experiences with primary caregivers during childhood [34]. The development of these early cognitive schemas (EMS) is crucial, as they determine how individuals perceive themselves, others, and the world, as well as how they relate to themselves and others. When early affective experiences are deficient, EMS are likely to develop, leading to maladaptive behavioral patterns and shaping the individual’s personality [35].

Numerous studies have shown that EMS are associated with a higher prevalence of personality disorders, depression, eating disorders, anxiety disorders, obsessive–compulsive disorder, and post-traumatic stress disorder [36]. EMS can be categorized into five domains: (1) Disconnection and Rejection, characterized by the expectation that others will not meet one’s needs for safety, acceptance, and respect; (2) Impaired Autonomy, linked to a negative perception of oneself and the environment concerning the capacity for success or independence; (3) Impaired Limits, associated with difficulties in establishing internal boundaries and assuming responsibilities toward others; (4) Other Directedness, encompassing schemas related to prioritizing the desires and emotions of others over one’s own; (5) Over-Vigilance and Inhibition, linked to the need for perfection and avoiding social disapproval [37,38].

Early maladaptive schemas are also associated with self-esteem. Dysfunctional schemas acquired during early childhood and solidified in adulthood tend to correlate with self-esteem difficulties [39,40,41]. Individuals with low self-esteem are generally less sociable, avoid new people and environments, show reduced kindness, and demonstrate limited communication, leading to overall social disconnection and increased social anxiety [42].

Furthermore, early maladaptive schemas are commonly linked to addictive pathologies, suggesting that these schemas may represent a common risk factor for both substance and behavioral addictions [43]. Specifically, concerning behavioral addictions, evidence has demonstrated the relationship between EMS and compulsive sexual behaviors [44,45], food addiction [46], pathological gambling [47], problematic internet use [48], social media addiction [49], and video gaming addiction [47]. However, relatively few studies have addressed the role of EMS in exercise addiction.

In systematic reviews [43,50] on early maladaptive schemas and behavioral addictions, only one study on exercise addiction was identified: the research conducted by Rankin et al. [51]. This study found that, in a sample of cyclists composed predominantly of men, EMS, particularly those within the domains of Other Directedness and Impaired Limits, accounted for a significant proportion of the variability in exercise addiction symptomatology. Overall, it appears that exercise addiction is more frequently detected in men than in women, with clear differences in the explanatory variables, highlighting the need for further investigation in this area [52]. Similarly, the incidence of exercise addiction seems to be higher among younger individuals, with an inverse relationship observed between exercise addiction and age [53,54,55].

Exercise addiction has been a topic of growing interest in the psychological and health literature due to its association with negative consequences for both physical and mental well-being [17,18,19,20,21]. However, most studies have focused on general populations or athletes, leaving a significant gap in understanding this phenomenon among young female university students. This group faces unique pressures, such as the internalization of thinness and muscularity ideals [56], the influence of social media on body perception [57], and the stress associated with academic performance and social expectations [58]. These pressures may exacerbate vulnerability to developing addictive exercise behaviors, particularly in the presence of EMS and fragile self-esteem [35]. This study aims to bridge this gap by exploring how these factors interact in female university students, providing a novel perspective that could inform more targeted and effective interventions to promote their overall well-being.

Given the scarcity of research addressing exercise addiction, self-esteem, and EMS, this study proposes novel and previously unexplored objectives: to analyze the relationship between exercise addiction and self-esteem; to investigate the association between exercise addiction and EMS; and to examine the potential mediating role of EMS in the relationship between exercise addiction and self-esteem. The study focuses on a sample of young women, specifically recruited from a university context to gain access to this particular population group.

Thus, one of the main objectives of this research is to explore the extent to which the negative effects of exercise addiction on self-esteem may be mediated by EMS. Our hypothesis proposes that exercise addiction may contribute to the activation of maladaptive early patterns, which, in turn, could negatively impact self-esteem. Based on this hypothesis, we employ mediation models in which the mediating variable between exercise addiction and self-esteem is be different types of EMS. Additionally, given the influence of certain sociodemographic variables on the variables of interest—particularly exercise behavior, self-esteem, and their relationship (e.g., gender, education level, and nationality) [59,60,61]—this study has been conducted on a homogeneous sample regarding these characteristics (Spanish female university students). However, this study also aims to analyze and control for the influence of other variables, such as age, employment status (employed/unemployed), and sexual orientation, whose impact on the relationship between exercise addiction and self-esteem has been less explored [62,63]. To achieve this, we first examine the relationship between these variables and the key variables of interest (exercise addiction, early maladaptive schemas, and self-esteem) through bivariate analyses. Subsequently, variables that show significant associations are included as covariates in the mediation models.

## 2. Materials and Methods

### 2.1. Sample and Design

This study is based on a non-experimental, correlational, and cross-sectional design. The sample was selected using non-probability sampling, recruiting students from the Complutense University of Madrid (Spain). Data collection was conducted through paper surveys administered in university classrooms. All participants provided informed consent before taking part in the study. The study data have been uploaded to Figshare and are available for anyone interested in consulting it. These data can be found here: Figshare; https://doi.org/10.6084/m9.figshare.28094177.

### 2.2. Ethical Approval, Informed Consent and Data Collection

This study adhered to the principles outlined in the Declaration of Helsinki [64]. Participants were informed of the aim of the study prior to providing their written informed consent to take part. They were also informed that they may withdraw from the study at any point. They were also informed that their responses would be kept completely anonymous and used only for research purposes. To ensure participants’ comfort, especially when addressing sensitive topics such as self-esteem or maladaptive schemas, they were asked to maintain a minimum physical distance of one seat (approximately one meter) between each other to maximize privacy. Additionally, they were requested to remain completely silent and refrain from discussing the questions or their responses with others to avoid disturbing or intimidating their peers.

This study was carried out in full accordance with the ethical guidelines governing psychological research. Authorization was granted by the Deontological Commission of the Faculty of Psychology at the Complutense University of Madrid (UCM), underscoring a dedication to maintaining the deontological principles set forth by the Official College of Psychologists and the Scientific Societies of Psychology. Conducted under reference number 2020/21-035, the study ensured strict adherence to all ethical protocols to protect the participants’ integrity and well-being.

During scheduled class sessions, students completed the questionnaires in their classrooms. They were assured that participation was voluntary, and if they chose not to participate, they could return a blank questionnaire without repercussion. Additionally, students were assured that their responses would remain confidential and anonymous, utilized solely for statistical analysis. No compensation was provided to the students for their participation. They were allotted between 20 and 30 min to complete the questionnaires. Informed consent was obtained from all subjects involved in this study.

### 2.3. Participants

Specifically, a minimum number of *n* = 120 was considered as the sample size for studies involving mediation in their analysis [65]. Specifically, the inclusion criteria were as follows: being 18 years of age or older, being enrolled in a university degree, being female, to engage in regular physical exercise (at least once a week), and not presenting any psychiatric comorbidity. A total of 788 university women finally participated in the study, constituting the final research sample.

It is considered relevant to include the sociodemographic variables of the participants collected in this study, such as age, socioeconomic status, and sexual orientation, as various studies have highlighted their influence on the relationship between exercise addiction and self-esteem. In this regard, previous research suggests that individuals with greater economic resources may have more opportunities to develop compulsive exercise patterns, while those in disadvantaged socioeconomic contexts may face barriers that affect their self-esteem and relationship with exercise [66].

Similarly, it has been found that age influences the relationship between exercise addiction and self-esteem, primarily through the reduction in exercise-dependence symptoms in older individuals and the greater emotional stability that can accompany aging [62]. Moreover, sexual orientation plays a role in this relationship for various reasons, such as belonging to sexual minorities [67]; social pressure and the search for acceptance [68]; body image concerns [69]; and experiences of discrimination, stigmatization, and the need to enhance self-esteem and regulate stress [70].

### 2.4. Measures

#### 2.4.1. Early Maladaptive Schemas

The Spanish adaptation [71,72,73] of the Cognitive Schema Questionnaire-Short Form-SQ-SF [74,75] was used. This is an instrument designed to assess people’s cognitive schemas. This instrument has proven to be a valid and reliable tool for assessing EMS, which is essential for understanding the psychological dynamics underlying exercise addiction. The Spanish version [71] ensures cultural and linguistic appropriateness for Spanish-speaking populations, which is crucial for guaranteeing the accuracy of results in this context. Additionally, its brief format makes it particularly suitable for university populations as it reduces administration time without compromising the depth of the assessment. Finally, its use in previous studies with similar populations supports its applicability and relevance in exploring how EMS influence self-esteem and addictive behaviors in young women [76,77,78].

It consists of 75 items; however, in this study, 65 items distributed in 13 scales have been used: (a) emotional deprivation, belief that the normal degree of emotional support will not be adequately met; (b) abandonment, belief that the person will be abandoned by loved ones; (c) abuse, belief that one will be abused, humiliated, cheated, or lied to by others; (d) failure, belief that one has failed or will inevitably fail; (e) dependency, belief that a person, without outside help from others, is not sufficient to cope with his or her responsibilities; (f) attachment, excessive involvement and emotional closeness with significant others (often parents); (g) subjugation, excessive subjection to the control of others, because one feels obliged, usually to avoid reactions of anger or abandonment; (h) emotional inhibition, excessive inhibition of actions and feelings usually to avoid the disapproval of others; (i) unattainable standards, hypercritical attitude towards oneself and others; (j) grandiosity, belief that one is superior to others, that one has special rights and privileges; (k) insufficient self-control, exaggerated emphasis on avoidance of discomfort, pain, conflict, confrontation, responsibility, and responsibility; (l) imperfection, feeling that one is flawed, undesirable, inferior; and (m) self-sacrifice, excessive and willful concentration on satisfying the needs of others. The items were answered on a Likert-type scale with 5 response options from 1 ‘totally untrue’ to 6 ‘describes me perfectly’. Cognitive schemas are organized into five main domains [37]:

1. Disconnection and rejection: this refers to schemas that involve the expectation that one’s own needs for security, acceptance, and respect will not be met by others. It includes schemas of emotional deprivation, abandonment, abuse/mistrust, social isolation, and imperfection.
2. Impaired Autonomy and Performance: These are schemas related to a negative view of oneself and the context in terms of one’s ability to succeed or to develop independence. It includes failure, dependency, vulnerability to danger, and attachment schemas.
3. Impaired Limits: This refers to encompasses schemas related to difficulties in setting internal limits and taking responsibility for others. It includes grandiosity schemas and insufficient self-control.
4. Other Directedness: This domain involves cognitive schemas related to satisfying the wishes and feelings of others. It includes subjugation, self-sacrifice, and recognition-seeking schemas.
5. Over-Vigilance and Inhibition: This refers to schemas related to the need to be perfect and to avoid disapproval from others. It includes the schemas of emotional inhibition, unattainable goals, negativity, and self-punitiveness.

In the present research, the 5 major domains are taken into account. The theoretical score for each domain ranges from 5 to 30. Higher scores imply more dysfunctional schemas. All scales achieved a Cronbach’s alpha greater than 0.70.

#### 2.4.2. Self-Esteem

The Rosenberg Self-Esteem Scale [79] was used to assess self-esteem given its extensive validation, reliability, and applicability in university populations [80]. This instrument is ideal for assessing global self-esteem in young women due to its brevity and ease of use. Furthermore, it has been widely employed in numerous studies linking self-esteem with addictive behaviors and psychological factors [81].

It consists of 10 items and a 4-option Likert-type response scale (from 1 ‘strongly disagree’ to 4 ‘strongly agree’) and is widely used internationally. Scores range from 10 to 40, with higher scores indicating higher self-esteem. It was validated in the Spanish population [82], and the version translated into Spanish [83] was used. It has good psychometric properties in terms of reliability and validity. Cronbach’s alpha in the present research was 0.87.

#### 2.4.3. Exercise Addiction

The Exercise Addiction Inventory (EAI) [84] was used. The Spanish adaptation [85] was applied with internal consistency values measured through a Cronbach’s alpha of 0.70 in our sample. This questionnaire was chosen for its brevity, reliability, and validation as a tool for assessing exercise addiction, widely used in young and university populations [86]. Its ability to identify addictive behaviors related to exercise makes it particularly suitable for exploring this phenomenon in female university students, as demonstrated in previous studies [87].

It is a self-report questionnaire consisting of 6 items with a 5-point Likert-type response format ranging from 1 ‘completely disagree’ to 5 ‘completely agree’. It assesses the risk of being addicted to physical exercise, using three cut-off points (24 points indicates risk of addiction, between 13 and 23 points suggests symptoms of addiction and less than 13 points indicates no symptoms).

### 2.5. Analytical Procedure

In the descriptive analysis of the sample, appropriate summary statistics were utilized. Categorical variables were presented using absolute (n) and relative (%) frequencies. Continuous variables were summarized with the mean and standard deviation. To assess the association between categorical variables, a Chi-squared test of independence was performed. To compare the distribution of continuous variables between two or more groups, T-tests and one-way ANOVA were employed. Pearson’s correlation was used to explore the relationship between two continuous variables.

For mediation analysis, SPSS macro PROCESS (model 4) was used. As recommended by Hayes [88], the regression/trajectory coefficients are all in non-standardized form since the standardized coefficients generally do not have a useful substantive interpretation. The tested model included Exercise Addiction as a predictor (X), Early Maladaptive Schemas (M) as a mediator, and self-esteem as a dependent variable (Y). Direct, indirect, and total effects were estimated. The direct path is the effect of a variable X on a variable Y. The indirect effect is the path linking X to Y via a mediator. The total effect of X on Y is the sum of the direct and indirect effects. To test the statistical significance of the indirect effects, percentile-based, bias-corrected (BC), and bias-corrected and accelerated (BCa) bootstrap confidence intervals (CIs) were computed [89,90]. The bootstrap estimates were based on 5000 bootstrap samples and a 95% CI was considered. All analyses were performed using SPSS version 28 [91].

## 3. Results

### 3.1. Socio-Demographic Characteristics of Participants

The sample consisted of a total of 788 women university students. All the women resided in Madrid (Spain) and were Spanish nationals. The age range was between 18 and 30 years old. Most of them (90%) were between 18 and 23 years old. The highest percentage was among 18-year-olds (23.5%), followed by 19-year-olds (20.6%), 20-year-olds (16.1%), 21-year-olds (13.5%), 22-year-olds (10.2%), and 23-year-olds (6.3%). The mean age was 20.39 and the standard deviation was 2.28. Among the participants, 15.2% were only children. The majority had one sibling (59.6%), 15.6% had two siblings, and 4.7% had three siblings. The rest of the sample (5%) had between four and six siblings.

In terms of cohabitation, 88.6% lived with their family, 2% with their partner, 4.6% with friends, and 4.9% lived alone.

All the participants were single. Regarding sexual orientation, 4.9% (n = 39) were homosexual, 78.8% (n = 621) were heterosexual, and 16.2% (n = 128) were bisexual. Approximately one tenth (12.2%; n = 97) combined their university studies with work.

### 3.2. Correlations Between Variables of Interest

Table 1 shows the descriptive data of the variables of interest, as well as the correlations between them. As can be seen in the table, self-esteem presents average values, and the average scores for physical exercise addiction are below the midpoint of the scale. Regarding the established cut-off points of exercise addition, it is observed that 53.8% present no symptoms (n = 424), 25.3% (n = 199) present symptoms of addiction, and 20.9% (n = 165) present risk of addiction. In relation to early non-adaptive schemas, mean scores are in all cases below the theoretical midpoint of the scale. The lowest values are observed in the Impaired Autonomy domain, while the highest are observed in the Other Directedness domain.

In terms of correlations, significant correlations are observed in all cases, except for the relationship between exercise addiction and impaired autonomy. Early maladaptive schemas maintain positive correlations with physical exercise addiction, and negative correlations with self-esteem. Negative correlations between self-esteem and physical exercise addiction are observed. All schemas have positive and significant correlations with each other. The weight of correlations deserves special mention. The correlations between exercise addiction and self-esteem are moderate. Overall, the correlations of self-esteem with early maladaptive schemas are significantly higher than those of physical exercise addiction with early maladaptive schemas. With regard to the former, it is worth highlighting the high relationship between self-esteem and Disconnection and Rejection schema, Impaired Autonomy schema, and Other Directedness schema.

### 3.3. Relationships Between Socio-Demographic Variables and Variables of Interest

Significant correlations were observed between age and the following variables: self-esteem (r = 0.083, *p* = 0.019), the Disconnection and Rejection domain (r = −0.193, *p* < 0.001), the Impaired Autonomy domain (r = −0.141, *p* = 0.001), the Impaired Boundaries domain (r = −0.089, *p* = 0.034), and the Other Directedness domain (r = −0.133, *p* = 0.002).

Regarding sexual orientation, significant differences were observed in the Disconnection and Rejection domain (F = 4.924, *p* = 0.008) and in the Other Directedness domain (F = 3.402, *p* = 0.034). Specifically, regarding the Disconnection and Rejection domain, the post-hoc contrasts reveal significant differences (*p* = 0.005) between heterosexuals (Mean = 9.88, SD = 3.75) and bisexuals (Mean = 11.44, SD = 5). Regarding the Other Directedness domain, the post-hoc tests do not show significant differences between the different groups (not significant post-hoc), with the differences closest to significance being those found between bisexual (Mean = 14.37, SD = 4.47) and heterosexual orientation (Mean = 13.20, SD = 4.02) (*p* = 0.066).

Regarding the employment situation, significant differences in the impaired autonomy domain (t = 2.383, *p* = 0.018) were found, with higher scores in students (Mean = 8.76, SD = 3.25) than in those who work (Mean = 7.81, SD = 2.53).

### 3.4. Mediation Analysis: Influence of Self-Esteem on Exercise Addiction Across Early Maladaptive Schemas

**Figure 1:**
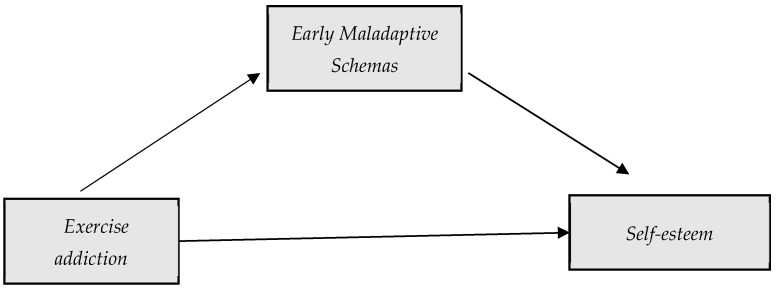
Proposed mediation model: exercise addiction–maladaptive schemas–self-esteem.

Figure 1 shows the theoretical model proposed. Therefore, five simple mediation models are proposed, each one in relation to one of the early maladaptive schemas.

**Figure 2:**
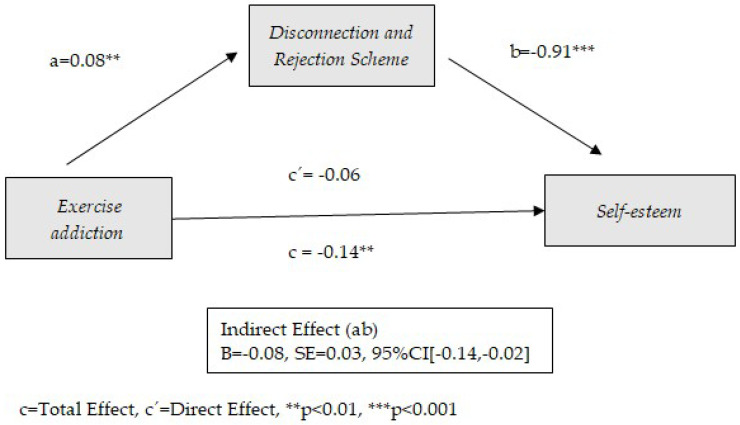
Model of mediation between exercise addiction and self-esteem through the use of Disconnection and Rejection schema.

**Figure 3:**
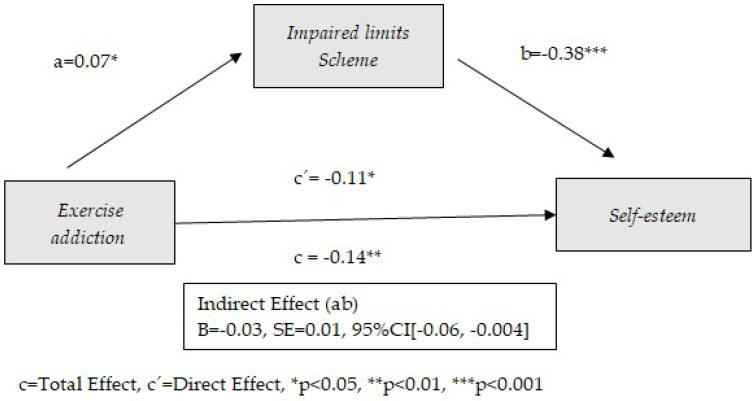
Model of mediation between exercise addiction and self-esteem through the use of Impaired Limits schema.

**Figure 4:**
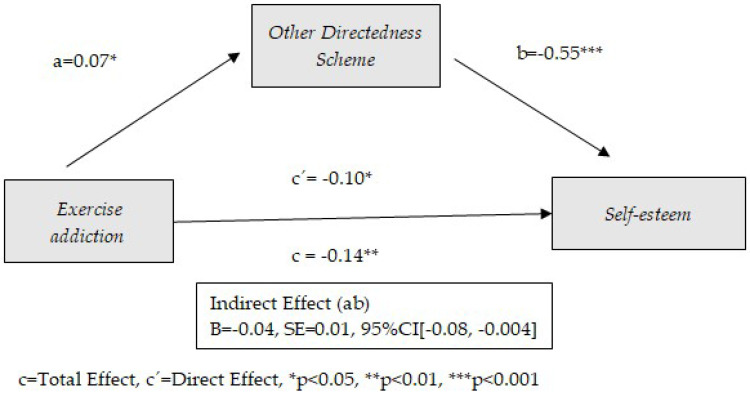
Model of mediation between exercise addiction and self-esteem through the use of Other Directedness schema.

**Figure 5:**
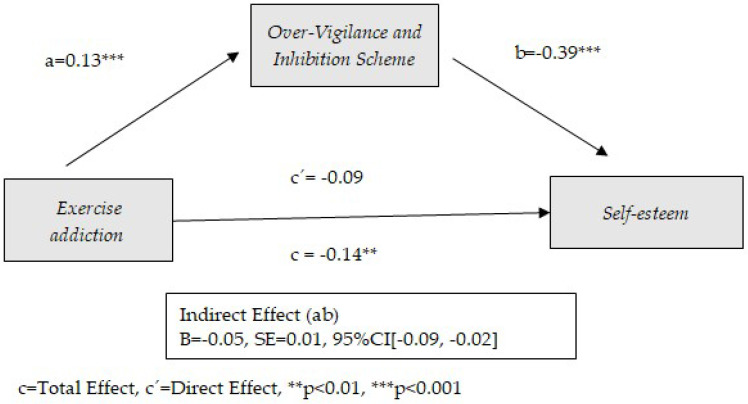
Model of mediation between exercise addiction and self-esteem through the use of Over-Vigilance and Inhibition schema.

The preconditions established for the mediation models (significant correlations between predictor and mediator, between mediator and consequent, and between predictor and consequent) show the possibility of mediation between exercise addiction and self-esteem for four of the early schemas considered: Disconnection and Rejection, Impaired Limits, Other Directedness, and Over-Vigilance and Inhibition. The following tables (Table 2, Table 3, Table 4 and Table 5) show the results obtained from these mediation models. Similarly, the graphical representation is presented in Figure 2, Figure 3, Figure 4 and Figure 5.

Table 2 shows the results of the effect of exercise addiction on self-esteem through Disconnection and Rejection as an early dysfunctional schema. As the results show, the Disconnection and Rejection schema plays a mediating role between exercise addiction and self-esteem. No direct effect is observed between exercise addiction and self-esteem (*p* = 0.103). Only age has a significant effect as a covariate (*p* = 0.004). The model contributes to 42% of the variance of self-esteem. Exercise addiction is positively related to the Disconnection and Rejection schema (B = 0.08, *p* = 0.008), which in turn contributes to a decrease in self-esteem (B = −0.91, *p* < 0.001). It is interesting to note the overall mediating role of the Disconnection and Rejection schema in the relationship between exercise addiction and self-esteem. In other words, the negative effect of physical exercise addiction on self-esteem is explained (in the model proposed) solely through the Disconnection and Rejection schema. Disconnection and rejection schemas are characterized by the belief that one’s needs for security, acceptance, and respect will not be fulfilled by others. These schemas encompass emotional deprivation, abandonment, mistrust/abuse, social isolation, and feelings of imperfection. The results indicate that physical exercise addiction in association with low self-esteem is produced through these schemas. The results are shown graphically in Figure 2.

Table 3 shows the results of the effect of exercise addiction on self-esteem through Impaired Limits as an early dysfunctional schema. As the results show, the Impaired Limits schema plays a mediating role between exercise addiction and self-esteem. In addition, direct effects between exercise addiction and self-esteem are observed (*p* = 0.017). Only age has a significant effect as a covariate (*p* < 0.001). The model contributes to 13% of the variance of self-esteem. Exercise addiction is positively related to the Impaired Limits schema (B = 0.07, *p* = 0.019), which in turn contributes to a decrease in self-esteem (B = −0.38, *p* < 0.001). In contrast to the Disconnection and rejection schema, the Impaired Limits schema plays a partial mediating role in the relationship between exercise addiction and low self-esteem; that is, although the Impaired Limits schema may contribute as an explanatory mechanism of the physical exercise–self-esteem relationship, it is also observed that this relationship is relatively independent of schema activation. Impaired Limits schemas involve difficulties in establishing personal boundaries and taking responsibility for others. These schemas include grandiosity and and a lack of self-control. The results are shown graphically in Figure 3.

Table 4 shows the results of the effect of exercise addiction on self-esteem through Other Directedness as an early dysfunctional schema. As the results show, the Other Directedness schema plays a mediating role between exercise addiction and self-esteem. In addition, direct effects between exercise addiction and self-esteem are observed (*p* = 0.024). Only age has a significant effect as a covariate (*p* < 0.001). The model contributes to 20% of the variance of self-esteem. Exercise addiction is positively related to the Other Directedness schema (B = 0.07, *p* = 0.032), which in turn contributes to a decrease in self-esteem (B = −0.55, *p* < 0.001). Similar to what was observed in the Impaired Limits schema, the Other Directedness schema plays a partial mediating role. In other words, the presence of the Other Directedness schema contributes as an intermediate mechanism to the relationship between physical exercise addiction and low self-esteem, but it is not essential. The Other Directedness schema focuses on meeting others’ needs, including subjugation, self-sacrifice, and recognition-seeking. The results are shown graphically in Figure 4.

Table 5 shows the results of the effect of exercise addiction on self-esteem through Over-Vigilance and Inhibition as an early dysfunctional schema. As the results show, the Over-Vigilance and Inhibition schema plays a mediating role between exercise addiction and self-esteem. Significance trends are observed in the direct effect between physical exercise addiction and self-esteem (*p* = 0.059). Only age has a significant effect as a covariate (*p* < 0.001). The model contributes to 14% of the variance of self-esteem. Exercise addiction is positively related to the Over-Vigilance and Inhibition schema (B = 0.13, *p* < 0.001), which in turn contributes to a decrease in self-esteem (B = −0.39, *p* < 0.001). The role of the Over-Vigilance and Inhibition schema in the relationship between exercise addiction and low self-esteem is very similar to that observed in relation to the Disconnection and Rejection schema. That is to say, it presents a total mediating role, being necessary for the exercise addiction–self-esteem relationship. However, taking into account the significance of the exercise addiction–self-esteem relationship (*p* = 0.059) and the contribution of Over-Vigilance and Inhibition schema to self-esteem (beta value = −0.39), it seems that the total mediating role of the Disconnection and Rejection schema has a greater magnitude than that of the Over-Vigilance and Inhibition schema. The Over-Vigilance and Inhibition schema involves striving for perfection and avoiding disapproval, including emotional inhibition, unattainable goals, negativity, and self-punishment. The results are shown graphically in Figure 5.

## 4. Discussion

The objectives of this study are to analyze the relationships between exercise addiction and self-esteem, explore the association of exercise addiction with EMS, and examine the potential mediating role of EMS in the relationship between exercise addiction and self-esteem. Firstly, the bivariate relationships in this study indicate that exercise addiction and self-esteem are negatively correlated, meaning that lower self-esteem is associated with higher levels of exercise addiction. These findings align with previous research in the literature [22,23,24,25,26]. However, other studies [92] found high self-esteem to be a predictor of exercise addiction in a sample of female university students when controlling for body shame. Additionally, self-esteem mediated the relationship between body shame and exercise addiction in this study. Similarly, another study on women engaged in CrossFit identified two distinct profiles among those exhibiting high exercise addiction: one characterized by elevated self-esteem and high admiration-related narcissism, and the other characterized by low self-esteem and elevated rivalry-related narcissism [93]. Moreover, Ref. [94] also found that high self-esteem is correlated with greater exercise addiction [94]. On the other hand, exercise addiction shows positive, albeit low, correlations with all EMS except for Impaired Autonomy. These findings are consistent with a recent meta-analysis [43], which demonstrated that all EMS were associated with behavioral addictions, particularly the Disconnection and Rejection domain. However, contrary to the findings of this study, they also reported a significant association with the Impaired Autonomy domain. Some authors [43] further noted that EMS were associated with substance addictions, suggesting that these schemas likely constitute a common risk factor for addictive disorders. Similarly, a systematic review [50] found that the Disconnection and Rejection domain was the most strongly associated with all addictive behaviors, followed by Impaired Limits.

It is worth noting that, in general, the correlations between self-esteem and early maladaptive schemas are significantly higher than those with exercise addiction, especially in the domains of Disconnection and Rejection, Impaired Autonomy and Performance, and Other Directedness. This is consistent because each of these EMS domains contributes to the fragility of self-esteem by generating dysfunctional beliefs about oneself and relationships with others [37,38]. People with these schemas presents in the domains of Disconnection and Rejection, Impaired Autonomy and Performance, and Other Directedness tend to view their self-worth as dependent on external approval or their ability to meet unrealistic expectations, which undermines their sense of self-confidence and self-esteem. Furthermore, these dysfunctional beliefs (especially these domains), which lead to harmful patterns of thinking, feeling, and behaving, negatively impact mental health by increasing the likelihood of experiencing greater addictive disorders [43,51], depression [95,96] suicide risk [97], anxiety [98], personality disorders [99], and obsessive–compulsive and stress-related disorders [100]. However, other domains, such as Impaired Limits and Over-Vigilance, are less related to self-esteem difficulties. The Impaired Limits and Over-Vigilance domains focus on aspects such as a lack of personal boundaries, impulsivity, perfectionism, and hypervigilance toward mistakes. These domains are more related to difficulties in emotional and behavioral regulation but do not necessarily involve globally negative self-esteem. In contrast, domains such as Disconnection and Rejection or Impaired Autonomy are more directly linked to self-esteem, as they involve a perception of being unlovable, unworthy, or incapable of functioning independently [75]. In this regard, it has been noted that Impaired Limits and Over-Vigilance are less associated with self-esteem because they focus more on externalized behaviors (e.g., lack of boundaries) or rigid coping strategies (e.g., perfectionism) [101]. Similarly, previous research suggests that the Impaired Limits and Over-Vigilance domains have a lesser impact on self-esteem compared to domains that involve a perception of rejection or failure [102]. Likewise, after examining the relationship between EMS and personality factors, it was found that the Impaired Limits and Over-Vigilance domains are more related to traits such as low conscientiousness and neuroticism, respectively, rather than self-esteem [76].

Likewise, it is observed that all early maladaptive schemas (except Impaired Autonomy) serve as mediators between exercise addiction and self-esteem. In the case of Disconnection and Rejection, the mediation is total; that is, the negative relationship between exercise addiction and self-esteem only occurs when these schemas are activated through exercise. This suggests that individuals who expect their needs for respect, care, security, and acceptance to remain unmet by others are more likely to experience self-esteem issues and exercise addiction. This can be explained through the theory proposed by Hausenblas et al. [103], which posits that exercise addiction can develop via two pathways. On one hand, there is the irrational belief that improving one’s appearance and/or changing exercise habits will lead to approval or meeting others’ expectations. On the other hand, the other pathway is characterized by engaging in physical exercise as a coping strategy for life stressors without setting appropriate boundaries or considering long-term consequences. It is also worth noting that this domain is the only one that has a clear total mediating effect. Furthermore, the variance in self-esteem explained when this domain is introduced as a mediator is significantly higher compared to other models, which also play a mediating role but only partially. These findings strongly underscore the importance of early maladaptive schemas, particularly those related to Disconnection and Rejection, in explaining the relationship between exercise addiction and low self-esteem. This is especially relevant when addressing discrepancies in the data regarding the presence of high or low self-esteem and exercise addiction [92,93,94], as these discrepancies could potentially be explained by early maladaptive schemas associated with Disconnection and Rejection. Therefore, early maladaptive schemas, especially those related to the domain of Disconnection and Rejection, play a crucial role in the interaction between exercise addiction and self-esteem. These schemas, which reflect expectations that needs for respect, care, security, and acceptance will not be met by others, act as total mediators in this relationship. This means that the negative connection between exercise addiction and low self-esteem only manifests when these schemas are activated through exercise. Individuals who hold these beliefs may resort to exercise compulsively, either seeking external approval or using it as a coping strategy to manage stress, without establishing appropriate boundaries. This dynamic reinforces exercise addiction and perpetuates self-esteem issues, highlighting the importance of addressing these schemas to understand and treat both exercise addiction and the difficulties associated with self-esteem.

In the case of Impaired Limits and Other Directedness, the mediation is partial; that is, there is a relationship between exercise addiction and (negative) self-esteem that is independent of the mediating role of the schemas. These results are consistent with previous research [51] which point out that these same domains accounted for a significant proportion of the variability in exercise-dependence symptomatology, suggesting that individuals who have an excessive external focus on the wants and needs of others and/or who are unable to establish adequate internal boundaries may be at greater risk of developing exercise-dependence symptomatology than those with lower levels of maladaptive schemas.

The results seem to suggest that an important explanatory variable for the effects of excessive physical exercise on decreased self-esteem may be due to early maladaptive schemas that are activated when physical exercise is performed (i.e., the schemas would be the ‘what for’ of physical exercise). According to Young et al. [75], when an individual’s early maladaptive schema is activated by a specific situation, certain addictive behaviors may be set in motion to cope with their negative thoughts or feelings; that is, addictive behavior, in this case physical exercise addiction, could be understood as a maladaptive coping strategy [43,103]. In this sense, one of the early maladaptive schemas that seem to be activated more (with respect to the rest) through physical exercise are those included in the domain of Over-Vigilance and Inhibition, in such a way that people with a greater need to be perfect and avoid the disapproval of others are at greater risk.

As Griffiths [104] points out, the development of addictive disorders is complex and influenced by multiple factors, with early maladaptive schemas being another part of the Complex Addiction Jigsaw. A key part of both substance and behavioral addictions appears to be these early maladaptive schemas, such that addictive potential is increased when Disconnection/Rejection, Impaired Autonomy and Performance, and Other Directedness schemas are specifically present [105]. To this complex puzzle is added the variable of self-esteem, which, as previously indicated, is related to the development of addictive disorders. This paper highlights the mediating role of early maladaptive schemas connecting physical exercise addiction with self-esteem and could be explained by motivational theories; that is, when the behavior (physical exercise) is guided by extrinsic motivations (such as satisfying others, avoiding certain negative consequences, gaining social recognition, etc.), the feeling of autonomy and competence is not satisfied, impacting the individual’s self-esteem, well-being, and personal growth [106]. Furthermore, it should be noted that different studies indicate that women differ from men in terms of motivation to exercise, being predominantly extrinsic in women [107,108,109].

The results of this study, which link EMS, self-esteem, and exercise addiction in young female university students, reveal both similarities and differences compared to research conducted in other cultural and demographic contexts. For instance, studies in Western populations [68] have highlighted how EMS—such as abandonment or imperfection—predispose individuals to addictive behaviors, including exercise addiction, which aligns with our findings. However, research in non-Western contexts [110], as in the case of Asia, suggests that cultural pressures toward thinness and academic achievement may exacerbate these dynamics, an aspect less pronounced in our sample. Additionally, studies in the United Kingdom [86] have reported a higher prevalence of exercise addiction among men, contrasting with our exclusively female sample, where self-esteem and EMS appear to play a more significant role. These differences underscore the importance of considering cultural and gender-related factors when interpreting results and designing targeted interventions.

This study has some limitations, such as the cross-sectional design, which restricts the scope of the conclusions, as it does not allow for causal inferences to be made. In this regard, the cross-sectional design prevents the establishment of causal relationships between early maladaptive schemas, self-esteem, and exercise addiction, limiting the ability to infer directionality or changes over time [111]. In addition, non-probability sampling was employed because of its convenience and applicability to a specific population. Although the sample collected can be considered adequate in size, it is important to keep in mind that this method may introduce biases and limit the generalizability of our findings to a wider population. Therefore, the results should be interpreted with caution, considering these limitations. On the other hand, the inclusion criteria established make it difficult to generalize to other populations since the sample consists exclusively of university women. However, the specificity of the analyzed population (female university students) and the sample size provide reliability to the results, taking into account these socio-demographic variables in particular. Furthermore, focusing on a female sample addresses the need highlighted by [52] to examine exercise addiction differentially between men and women. Other limitations include the assessment tools, as they rely on self-reported measures and are used as screening tools without diagnostic validity [112]. Similarly, self-report questionnaires may be subject to social desirability bias, which could have influenced participants’ responses regarding their self-esteem and exercise behaviors, potentially leading them to underestimate problems or exaggerate achievements [113].

The results of this research emphasize the scarcity of previous research analyzing the relationship between early maladaptive schemas, self-esteem, and exercise addiction, particularly in women, and underline the need to take into account individual variables of the person (such as early maladaptive schemas) when working with people with physical exercise addiction. Although there is not a large scientific body on treatments for physical exercise addiction, cognitive behavioral therapy is proposed as a therapy of choice [114,115], and it is interesting to assess the intervention in maladaptive cognitive schemas and consider self-esteem as another variable to intervene [51]. Furthermore, it would be beneficial to develop educational programs in university and sports settings that promote intrinsic motives for exercise, such as well-being and enjoyment, rather than the pursuit of external validation or obsession with body image [116]. These programs should adopt a gender-sensitive approach to target specific at-risk groups of women. Likewise, the fundamental motivation for physical exercise should be assessed as it seems that women who exercise for health reasons tend to have better self-esteem [16,51]. This is supported by previous work that identifies intrinsic reasons for performing a behavior as enhancing self-esteem by facilitating physical exercise through enjoyable activities that provide a sense of pleasure and personal achievement [117,118]. The dissemination of these results is especially necessary among athletes (especially professional endurance athletes because they are at higher risk of exercise addiction, but also among amateurs); sports coaches and gyms or sports clubs; and psychologists and other professionals who are in contact with the group at risk of exercise addiction (especially in high-risk groups such as endurance athletes and women who engage in sports either recreationally or professionally). These interventions could be integrated into university counseling services and student wellness programs, thereby expanding their reach and applicability.

## 5. Conclusions

This study provides significant evidence regarding the relationship between exercise addiction, self-esteem, and early maladaptive schemas in young female university students. The results confirm that exercise addiction is negatively correlated with self-esteem, suggesting that women with lower self-esteem are more likely to develop addictive behaviors related to exercise. Furthermore, it is observed that EMS, particularly those related to the domain of Disconnection and Rejection, play a key mediating role in this relationship, explaining a significant proportion of the variability in self-esteem. These findings support the theory that exercise addiction may function as a maladaptive coping strategy to manage negative emotions associated with maladaptive schemas, such as the need for approval or fear of rejection.

In contrast to previous studies that have found a positive correlation between self-esteem and exercise addiction in certain contexts, our research suggests that EMS could be an underlying explanatory factor that clarifies these discrepancies. For example, while some women may use exercise as a way to improve their self-esteem, others may fall into addictive patterns due to the activation of EMS related to disconnection and rejection. This underscores the importance of addressing both EMS and self-esteem in therapeutic interventions, particularly for young women who face unique social and cultural pressures.

The practical implications of these findings are relevant for the design of interventions based on cognitive behavioral therapy (CBT), which could incorporate techniques to modify EMS and strengthen self-esteem. Additionally, the implementation of educational programs in university and sports settings is recommended to promote intrinsic motivations for exercise, such as well-being and enjoyment, rather than the pursuit of external validation.

Finally, this study highlights the need for future research using longitudinal designs and multimodal assessment methods to overcome the limitations associated with self-report questionnaires and cross-sectional designs. Expanding research to different cultural and demographic contexts would also allow for a deeper understanding of how EMS and self-esteem interact in exercise addiction, thereby contributing to more effective and targeted interventions.

## Figures and Tables

**Table 1:** Descriptive data and correlations between variables of interest.

		R^2^ (p)	R^2^ (p)	R^2^ (p)	R^2^ (p)	R^2^ (p)	R^2^ (p)
	Mean (SD)	(2)	(3)	(4)	(5)	(6)	(7)
(1) Self-esteem	26.78 (2.56)	−0.33 (<0.001)	−0.64 (<0.001)	−0.62 (<0.001)	−0.29 (<0.001)	−0.42 (<0.001)	−0.31 (<0.001)
(2) Exercise addiction	13.97 (7.83)		0.12 (<0.01)	0.08 (0.06)	0.11 (0.01)	0.10 (0.01)	0.16 (<0.001)
(3) Disconnection and Rejection	10.09 (3.99)			0.63 (<0.001)	0.43 (<0.001)	0.60 (<0.001)	0.43 (<0.001)
(4) Impaired autonomy	8.63 (3.18)				0.44 (<0.001)	0.53 (<0.001)	0.32 (<0.001)
(5) Impaired Limits	12.44 (3.91)					0.33 (<0.001)	0.28 (<0.001)
(6) Other Directedness	13.31 (4.10)						0.37 (<0.001)
(7) Over-Vigilance and Inhibition	12.90 (4.28)						

**Table 2:** Mediation model: Regression of exercise addiction on self-esteem through maladaptive schemas (Disconnection and Rejection) as mediator (n = 788).

Regression of Exercise Addiction on Self-Esteem Through Disconnection and Rejection				
Outcome variable: Disconnection and Rejection				
Exercise addiction	B (SE)	t	*p*	[LLCI-ULCI]
	0.08 (0.03)	2.64	0.008	[0.022/0.153]
Outcome variable: Self-esteem				
	B (SE)	t	*p*	[LLCI-ULCI]
Exercise addiction	−0.06 (0.03)	−1.63	0.103	[−0.139/0.012]
M: Disconnection and Rejection	−0.91 (0.05)	−18.64	<0.001	[−1.01/−0.81]
Covariate: Age	0.23 (0.08)	2.82	0.004	[0.073/0.405]
Covariate: Sexual orientation	0.15 (0.47)	0.326	0.744	[−0.769/1.07]
Covariate: Working	0.27 (0.51)	0.536	0.591	[−0.729/1.27]
Model summary R = 0.65	R^2^ = 0.42	F = 82.25	*p* < 0.0001	
Total Effect of X on Y	Eff. (SE)	t	*p*	[LLCI-ULCI]
	−0.14 (0.05)	−2.97	0.003	[−0.239/−0.047]
Direct effect of X on Y	Eff. (SE)	t	*p*	[LLCI-ULCI]
	−0.06 (0.03)	−1.63	0.103	[−0.139/0.012]
Indirect Effect of X on Y	Eff. (SE)			[LLCI-ULCI]
Disconnection	−0.08 (0.03)			[−0.139/−0.023]

**Table 3:** Mediation model: Regression of exercise addiction on self-esteem through Maladaptive Schemas (Impaired Limits) as mediator (n = 788).

Regression of Exercise Addiction on Self-Esteem Through Impaired Limits				
Outcome variable: Impaired Limits				
Exercise addiction	B (SE)	t	*p*	[LLCI-ULCI]
	0.07 (0.03)	2.35	0.019	[0.012/0.143]
Outcome variable: Self-esteem				
	B (SE)	t	*p*	[LLCI-ULCI]
Exercise addiction	−0.11 (0.04)	−2.38	0.017	[−0.20/−0.02]
M: Impaired Limits	−0.38 (0.06)	−6.41	<0.001	[−0.50/−0.27]
Covariate: Age	0.46 (0.10)	4.52	<0.001	[0.26/0.66]
Covariate: Sexual orientation	−0.50 (0.57)	−0.87	0.382	[−1.63/0.62]
Covariate: Working	0.50 (0.62)	0.799	0.424	[−0.730/1.73]
Model summary R = 0.36	R^2^ = 0.13	F = 16.69	*p* < 0.0001	
Total Effect of X on Y	Eff. (SE)	t	*p*	[LLCI-ULCI]
	−0.14 (0.05)	−2.97	0.003	[−0.239/−0.047]
Direct effect of X on Y	Eff. (SE)	t	*p*	[LLCI-ULCI]
	−0.11 (0.04)	−2.38	0.017	[−0.206/−0.019]
Indirect Effect of X on Y	Eff. (okSE)			[LLCI-ULCI]
Impaired Limits	−0.03 (0.01)			[−0.060/−0.004]

**Table 4:** Mediation model: Regression of exercise addiction on self-esteem through maladaptive schemas (Other Directedness) as mediator (n = 788).

Regression of Exercise Addiction on Self-Esteem Through Other Directedness				
Outcome variable: Other Directedness				
Exercise addiction	B (SE)	T	*p*	[LLCI-ULCI]
	0.07 (0.03)	2.14	0.032	[0.006/0.142]
Outcome variable: Self-esteem				
	B (SE)	T	*p*	[LLCI-ULCI]
Exercise addiction	−0.10 (0.05)	−2.26	0.024	[−0.19/−0.013]
M: Other Directedness	−0.55 (0.05)	−10.05	<0.001	[−0.66/−0.44]
Covariate: Age	0.39 (0.09)	4.04	<0.001	[0.204/0.59]
Covariate: Sexual orientation	−0.16 (0.55)	−0.292	0.769	[−1.24/0.92]
Covariate: Working	0.55 (0.59)	0.922	0.356	[−0.622/1.72]
Model summary R = 0.45	R^2^ = 0.20	F = 29.52	*p* < 0.0001	
Total Effect of X on Y	Eff. (SE)	T	*p*	[LLCI-ULCI]
	−0.14 (0.05)	−2.92	0.003	[−0.239/−0.047]
Direct effect of X on Y	Eff. (SE)	T	*p*	[LLCI-ULCI]
	−0.10 (0.04)	−2.25	0.024	[−0.191/−0.013]
Indirect Effect of X on Y	Eff. (SE)			[LLCI-ULCI]
Other Directedness	−0.04 (0.01)			[−0.079/−0.004]

**Table 5:** Mediation model: Regression of exercise addiction on self-esteem through maladaptive schemas (Over-Vigilance and Inhibition) as mediator (n = 788).

Regression of Exercise Addiction on Self-Esteem Through Over-Vigilance and Inhibition				
Outcome variable: Over-Vigilance and Inhibition				
Exercise addiction	B (SE)	t	*p*	[LLCI-ULCI]
	0.13 (0.03)	3.75	<0.001	[0.065/0.207]
Outcome variable: Self-esteem				
	B (SE)	t	*p*	[LLCI-ULCI]
Exercise addiction	−0.09 (0.05)	−1.88	0.059	[−0.182/0.003]
M: Over-Vigilance & Inh.	−0.39 (0.05)	−7.23	<0.001	[−0.50/−0.28]
Covariate: Age	0.47 (0.10)	4.66	<0.001	[0.274/0.672]
Covariate: Sexual orientation	−0.52 (0.56)	−0.916	0.359	[−1.64/0.596]
Covariate: Working	0.56 (0.62)	0.916	0.359	[−0.651/1.78]
Model summary R = 0.38	R^2^ = 0.14	F = 19.07	*p*< 0.0001	
Total Effect of X on Y	Eff. (SE)	t	*p*	[LLCI-ULCI]
	−0.14 (0.05)	−2.92	0.003	[−0.239/−0.047]
Direct effect of X on Y	Eff. (SE)	t	*p*	[LLCI-ULCI]
	−0.09 (0.04)	−1.88	0.059	[−0.182/0.003]
Indirect Effect of X on Y	Eff. (SE)			[LLCI-ULCI]
Over-Vigilance and Inhibition	−0.05 (0.01)			[−0.086/−0.024]

## Data Availability

The original contributions presented in the study are publicly available. This data can be found here: Figshare; https://doi.org/10.6084/m9.figshare.28094177.
